# Establishment of human hematopoietic organoids for evaluation of hematopoietic injury and regeneration effect

**DOI:** 10.1186/s13287-024-03743-y

**Published:** 2024-05-04

**Authors:** Keyi Chen, Yunqiao Li, Xumin Wu, Xuan Tang, Bowen Zhang, Tao Fan, Lijuan He, Xuetao Pei, Yanhua Li

**Affiliations:** 1 College of Chemistry & Materials Science, Hebei University, Hebei, Baoding, 071002 China; 2 Key Laboratory of Medicinal Chemistry and Molecular Diagnosis, Hebei University, Hebei, Baoding, 071002 China; 3 Stem Cell and Regenerative Medicine Lab, Beijing Institute of Radiation Medicine, Beijing, 100850 China

**Keywords:** Hematopoietic stem/progenitor cells, Hematopoietic organoids, Gelatin-methacryloyl, Radiation injury, Granulocyte colony-stimulating factor

## Abstract

**Background:**

Human hematopoietic organoids have a wide application value for modeling human bone marrow diseases, such as acute hematopoietic radiation injury. However, the manufacturing of human hematopoietic organoids is an unaddressed challenge because of the complexity of hematopoietic tissues.

**Methods:**

To manufacture hematopoietic organoids, we obtained CD34^+^ hematopoietic stem and progenitor cells (HSPCs) from human embryonic stem cells (hESCs) using stepwise induction and immunomagnetic bead-sorting. We then mixed these CD34^+^ HSPCs with niche-related cells in Gelatin-methacryloyl (GelMA) to form a three-dimensional (3D) hematopoietic organoid. Additionally, we investigated the effects of radiation damage and response to granulocyte colony-stimulating factor (G-CSF) in hematopoietic organoids.

**Results:**

The GelMA hydrogel maintained the undifferentiated state of hESCs-derived HSPCs by reducing intracellular reactive oxygen species (ROS) levels. The established hematopoietic organoids in GelMA with niche-related cells were composed of HSPCs and multilineage blood cells and demonstrated the adherence of hematopoietic cells to niche cells. Notably, these hematopoietic organoids exhibited radiation-induced hematopoietic cell injury effect, including increased intracellular ROS levels, γ-H2AX positive cell percentages, and hematopoietic cell apoptosis percentages. Moreover, G-CSF supplementation in the culture medium significantly improved the survival of HSPCs and enhanced myeloid cell regeneration in these hematopoietic organoids after radiation.

**Conclusions:**

These findings substantiate the successful manufacture of a preliminary 3D hematopoietic organoid from hESCs-derived HSPCs, which was utilized for modeling hematopoietic radiation injury and assessing the radiation-mitigating effects of G-CSF in vitro. Our study provides opportunities to further aid in the standard and scalable production of hematopoietic organoids for disease modeling and drug testing.

**Supplementary Information:**

The online version contains supplementary material available at 10.1186/s13287-024-03743-y.

## Introduction

In recent years, various tissue-specific organoids have been generated in vitro based on their capacity for stem cell proliferation, differentiation, and self-organization within a three-dimensional (3D) microenvironment [1–3]. Organoid culture techniques have been continuously developed and applied in disease modeling, drug candidate screening, and investigation of pathophysiological mechanisms [4]. Given the important potential applications of hematopoietic organoids and organ-on-a-chip systems, several laboratories are dedicated to the manufacturing and assessment of their utility in disease modeling, particularly for conditions such as radiation injury and bone marrow (BM) fibrosis [1, 5]. Nevertheless, establishing a reliable culture technique for hematopoietic organoids remains a formidable challenge owing to the intrinsic complexity of hematopoietic tissues.

Hematopoietic stem cells (HSCs), situated at the apex of the hematopoietic system, have the ability to self-renew and differentiate into multilineage blood cells [6], which are critical seed cells for manufacturing of hematopoietic organoids. However, enriching rare adult HSCs and their progenitor cells (HSPCs) in the BM is a significant challenge. The limited number and expansion capacity of HSPCs from a single cord blood sample also impede the development of large numbers of homogeneous hematopoietic organoids. Human pluripotent stem cells (hPSCs), including induced pluripotent stem cells (iPSCs) and human embryonic stem cells (hESCs), have indefinite capacity for self-renewal and the potential to differentiate into HSPCs [7]. hPSCs-derived HSPCs may be ideal seed cells for generating human hematopoietic organoids [8–10]. In vivo, adult HSPCs inhabit a specialized BM niche comprising a variety of stromal cells, such as mesenchymal cells, endothelial cells, osteocytes, and the extracellular matrix (ECM) [11–13], which collectively provide intricate biochemical and physical cues essential for the regulation and maintenance of HSPC function. To construct a biomimetic bone marrow microenvironment in vitro, multiple cell lines, such as bone marrow stromal cells (HS-5), human umbilical vein endothelial cells (HUVECs) or human umbilical artery endothelial cells (HUAECs), and osteoblasts (hFOB 1.19) have been used in culture systems to support HSPC survival, proliferation, and differentiation [14–17]. The 3D scaffold is another important factor in mimicking the hematopoietic microenvironment. In recent years, gelatin-methacryloyl (GelMA) hydrogels have become an alternative scaffold material for constructing a 3D microenvironment or acting as a cell-laden bioprinting bio ink, owing to their good biocompatibility and tunable physicochemical properties [18]. It is important to investigate whether human hematopoietic organoids can be manufactured with GelMA and multiple niche-related cells, and whether these organoids can be used as a disease model, such as a radiation-injured model, to evaluate drug response.

In this study, we proposed a method for constructing hematopoietic organoids using a GelMA-based 3D culture system with HSPCs derived from hESCs. To better mimic the bone marrow microenvironment, we incorporated niche-related cells such as HS-5 cells, HUAECs, and hFOB 1.19 cells into the GelMA. Our data suggests that the presence of these niche cells creates a more favorable microenvironment for the formation of hematopoietic organoids. Furthermore, our findings demonstrate that these hematopoietic organoids display radiation-induced injury to hematopoietic cells and show responsiveness to granulocyte colony-stimulating factor (G-CSF). This study contributes to the advancement of standardized and scalable production methods for hematopoietic organoids, which can greatly facilitate their utilization in disease modeling and drug testing.

## Materials and methods

### hESC culture and differentiation

H9 human embryonic stem cells (H9-hESCs; WiCell Research Institute) were maintained on Matrigel (CORNING, Corning, NY, USA) and cultured in mTeSR1 medium (STEMCELL Technologies, Vancouver, Canada). The medium was refreshed daily, and the cells were passaged at 4–6 day intervals using TrypLE Select (Thermo Fisher Scientific, Waltham, MA, USA). On Day 0, hESCs were plated at a density of 1 × 10^4^ cells/cm^2^ and cultured for 24 h in the mTeSR medium. The following day (day 1), the medium was replaced with BEL medium supplemented with 25 ng/ml bFGF (PeproTech, Rocky Hill, NJ, USA), 25 ng/ml BMP4, 25 ng/ml Activin A (both from R&D Systems, Minneapolis, MN, USA), and 2 µM CHIR99021 (Selleck, Houston, TX, USA), and the cells were cultured for an additional 48 h (Stage I). Afterwards, cells were rinsed with phosphate-buffered saline (PBS) and transitioned to Stage II BEL medium, enhanced with 50 ng/ml VEGF (R&D Systems), 20 ng/ml bFGF (Selleck), and 2 µM SB431542 (Selleck), and incubated for 72 h. After incubation, the cells were dissociated into single-cell suspensions. CD34^+^ cells were then isolated by positive selection using a MACS CD34 MicroBead Kit (Miltenyi Biotec, Gladbach Bergisch, Germany) [19–21]. Isolated CD34^+^ cells were then resuspended in BEL medium containing 50 ng/mL SCF (PeproTech), 20 ng/mL TPO (PeproTech), 20 ng/mL IL-3 (PeproTech), 20 ng/mL Flt3L (PeproTech), 20 ng/ml VEGF, 10 ng/mL bFGF, and 5 µM SB431542. Cells were seeded at a density of 2 × 10^4^ cells/cm^2^ in Matrigel-coated culture dishes and cultured for 96 h. At the end of this period, the supernatants were harvested, and CD34^+^ hematopoietic progenitor cells were further purified using the MACS CD34 MicroBead Kit. The BEL medium composition followed a published reference (see Additional file 2: Table S1) [22], and the main reagents utilized for hESCs maintenance and differentiation are outlined in Additional file 4: Table S3 and Additional file 5: Table S4.

### Stromal cell culture

HS-5 cells (ATCC) were cultured in Dulbecco's Modified Eagle Medium (DMEM) supplemented with 10% fetal bovine serum (FBS) (both from Thermo Fisher Scientific) at 37 °C. The medium was refreshed every two days, and the cells were passaged every 3–4 days using trypsin–EDTA [23].

HUAECs were cultured in EGM-2 medium (Lonza, Basel, Switzerland) at 37 °C [16]. The medium was refreshed every two days, and the cells were passaged every 3–4 days using TrypLE Select.

hFOB 1.19 cell line (Cell Bank/Stem Cell Bank of the Chinese Academy of Sciences) was cultured in DMEM/F12 medium supplemented with 10% FBS and 0.3 mg/ml G418 (Thermo Fisher Scientific) at 33.5°. The medium was refreshed every two days, and the cells were passaged every 4–5 days using TrypLE Select [14].

### Preparation and cell culture of GelMA hydrogel

GelMA (EFL, Suzhou, China) was solubilized in a PBS solution containing 0.25% (w/v) lithium phenyl-2,4,6-trimethylbenzoylphosphinate (LAP; EFL) to attain a final GelMA concentration of 10% (w/v). The mixture was heated to 85 °C in a water bath shielded from light for 45 min and subsequently sterile-filtered.

For co-culture experiments, CD34^+^ cells were labeled with 2 µM carboxyfluorescein succinimidyl ester (CFSE) dye (Thermo Fisher Scientific) [20].

HS-5 cells, HUAECs, and hFOB 1.19 cells and hESCs-derived CD34^+^ cells were seeded into GelMA hydrogel at a ratio of 1:1:1:3 to achieve a final density of 1 × 10^6^ cells/mL for CD34^+^ cells. The GelMA and cell mixture was then deposited in ultra-low attachment 24-well plates and polymerized using UV light for 51 s. In the GelMA hydrogel group, CD34^+^ cells pre-labeled with CFSE dye were encapsulated in GelMA at a density of 1 × 10^6^ cells/mL. Both groups were cultured in a 1:1 mixture of BEL and EGM-2 media, respectively. The BEL medium was supplemented with 50 ng/mL SCF, 20 ng/mL TPO, 20 ng/mL IL-3, 20 ng/mL Flt3L, 5 ng/mL bFGF, and 5 µM SB431542. All cell cultures were maintained at 37 °C with 5% CO_2_, with media refreshment every 2 days.

### Suspension culture of HSPCs

For the suspension culture of CD34^+^ cells, an equal number of CFSE-stained CD34^+^ cells were plated in 6-well plates, corresponding to the amounts utilized in both the GelMA hydrogel co-culture and isolated CD34^+^ cell cultures. The cells were subsequently incubated at 37 °C with 5% CO_2_ in a mixed medium composed of BEL and EGM-2 media at a 1:1 ratio. The BEL medium was fortified with 50 ng/mL SCF, 20 ng/mL TPO, 20 ng/mL IL-3, 20 ng/mL Flt3L, 5 ng/mL bFGF, and 5 µM SB431542. Throughout the cultivation period, the medium was refreshed or replaced daily.

### Flow cytometry analysis

For the analysis of cell surface antigens, cells were harvested using GelMA Lysis Buffer (EFL), rinsed once with PBS, and subsequently incubated with specific antibodies diluted in PBS for 30 min at 4 °C in darkness. Following staining, the cells were examined using a BD FACS Aria II flow cytometer (BD Biosciences, Franklin Lakes, NJ, USA), and the data were processed using FlowJo software (TreeStar, Ashland, OR, USA) [24, 25]. The antibody details are provided in Additional file 2: Table S2.

### Immunofluorescence assay

For the 3D cell-staining experiment, GelMA laden with cells was fixed with 4% paraformaldehyde at room temperature for one hour. Subsequently, the samples were permeabilized with 0.3% Triton X-100 in PBS for 30 min and blocked with 10% donkey serum in PBS for two hours. Primary antibodies were applied in 10% donkey serum and incubated overnight at 4 °C, washed three times for 1 h each. The secondary antibodies were incubated overnight at room temperature and subjected to the same washing steps. The nuclei were stained with 10 µg/ml DAPI for 15 min. Fluorescence microscopy images were captured using a confocal microscope (PerkinElmer, Waltham, MA, USA) [24, 26]. Detailed information on the antibodies used is provided in Additional file 2: Table S2.

### ELISA assay

For the ELISA assay, cells were collected using GelMA Lysis Buffer. The levels of CXCL12 in the cells were detected by using Human CXCL12 ELISA Kit (BOSTER, Wuhan, China) according to the manufacturer’s instructions.

### Colony-forming unit assay

For the hematopoietic colony-forming unit (CFU) assay, cells were collected using GelMA Lysis Buffer and fixed at a density of 5 × 10^5^ cells/mL. A 100 μL cell suspension was then combined with 0.5 mL of H4636 hematopoietic colony culture medium (STEMCELL Technologies), thoroughly mixed, and dispensed into ultra-low attachment 24-well plates. After 14 days of incubation, colonies were enumerated and classified based on morphology into erythroid (BFU-E, burst-forming unit-erythroid), myeloid (CFU-GM, colony-forming unit-granulocyte, monocyte), or mixed lineage (CFU-GEMM, colony-forming unit granulocytes, erythrocytes, monocytes, and megakaryocytes) categories [21].

### G‑CSF administration after radiation

Immediately after exposure to 2 or 4 Gy radiation, the medium was replaced with a mixture of BEL and EGM-2 (as previously described) supplemented with 100 ng/mL G-CSF. The culture was maintained by daily replacement with fresh medium containing 100 ng/mL G-CSF.

### Statistical analysis

Statistical analyses were conducted using GraphPad Prism 8 software (GraphPad, Inc., La Jolla, CA, USA). Data are presented as means ± SD. The statistical significance of the differences was assessed using an unpaired two-tailed Student’s t-test, one-way ANOVA, or two-way ANOVA, as appropriate. Statistical significance was considered at *p* < 0.05, denoted as *p < 0.05, **p < 0.01 and ***p < 0.001.

## Result

### GelMA hydrogel is capable of maintaining undifferentiated state of hESCs-derived HSPCs

**Fig. 1:**
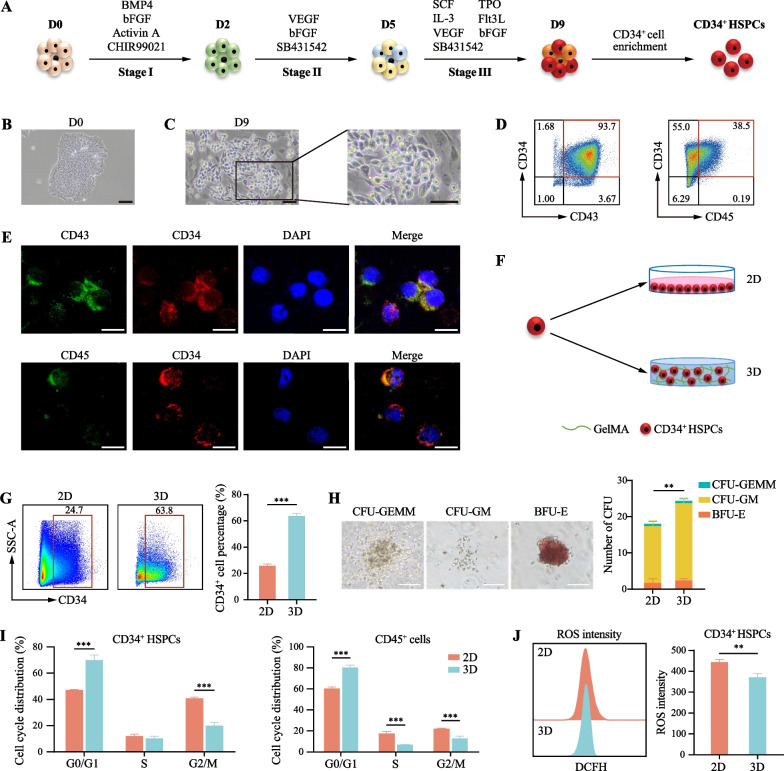
Establishment of a hESC-to-HSPC differentiation system and the application of GelMA in vitro culture. **a** Schematic diagram of the differentiation from hESCs to HSPCs. **b** Representative morphology of hESCs-H9 cells (scale bar, 100 µm). **c** Representative morphologies of HSPCs induced from hESCs-H9 cells (scale bar, 50 µm). **d** Flow cytometry analysis of the expression of CD34, CD43, and CD45 on the induced HSPCs. **e** Immunofluorescence staining for the detection of CD34, CD43, and CD45 expression on the induced HSPCs (scale bar, 20 µm). **f** Schematic diagram of 2D and 3D-GelMA culture. **g** Percentage of CD34^+^ cells on day 7 for each condition. Results are presented as the mean ± SD from three independent experiments. Unpaired Student’s *t*-test, ***p < 0.001. **h** Representative morphologies of different colony types (CFU-GEMM, CFU-GM and BFU-E) and total number of colonies under different culture conditions (scale bar, 100 µm). Results are presented as the mean ± SD from three independent experiments. Unpaired Student’s *t*-test, **p < 0.01. **i** Percentage of CD34^+^ and CD45^+^ cells in G0/G1, S and G2/M phase of the cell cycle for each condition after 7-day culture. Results are presented as the mean ± SD from three independent experiments. Two-way ANOVA, ***p < 0.001. **j** Intracellular ROS levels within CD34^+^ cells were determined utilizing DCFH assay via flow cytometry. Results are presented as the mean ± SD from three independent experiments. Unpaired Student’s *t*-test, **p < 0.01

hESCs are ideal seed cells for generation of large numbers of HSPCs for hematopoietic organoid construction. Thus, we first opted for an adherent induction protocol to produce mesodermal progenitors, hemogenic endothelial cells, and HSPCs from hESCs within a 9-day induction period (Fig. 1a). Cultured hESCs were orderly exposed to a three-stage differentiation culture medium, resulting in the gradual emergence of semi-adherent and adherent hematopoietic-like cells (Fig. 1b, c). Subsequently, populations of hematopoietic-like cells were selectively enriched using CD34^+^ immunomagnetic bead sorting [21]. Flow cytometry analysis revealed high expression levels of CD43, CD34, and CD45 in these cells, with CD43^+^CD34^+^ cells comprising approximately 93.70% and CD34^+^CD45^+^ cells constituting approximately 38.50% of the population (Fig. 1d). Additionally, the presence of CD34, CD43, and CD45 proteins was confirmed by immunofluorescence staining (Fig. 1e), providing further evidence of the successful collection of HSPCs from hESCs.

To construct hematopoietic organoids, GelMA hydrogel was selected as the 3D scaffold into which hESCs-derived CD34^+^ HSPCs were seeded (Fig. 1f) [26–29]. Interestingly, we found that the CD34^+^ cell percentage was significantly higher in the 3D GelMA culture microenvironment than in the two-dimensional (2D) liquid culture condition (Fig. 1g). Colony-forming unit (CFU) assay results revealed that the total colony count markedly increased in the 3D GelMA culture group after a 7-day culture (Fig. 1h). These results suggested that the GelMA hydrogel more effectively maintained the proportion of hESCs-derived HSPCs in vitro (Fig. 1g). Furthermore, we examined the cell cycles of CD34^+^ and CD45^+^ cells under 2D and 3D GelMA culture conditions. We found that hESCs-derived CD34^+^ and CD45^+^ hematopoietic cells presented lower cell percentages in the G2/M/S phase and higher percentages in the G0/G1 phase when cultured in 3D conditions (Fig. 1i). Additionally, lower levels of ROS were detected in hESCs-derived CD34^+^ HSPCs in the 3D GelMA culture (Fig. 1j), suggesting that the GelMA hydrogel may maintain the undifferentiated state of HSPCs by reducing ROS levels and maintaining more cells in a resting state. These indicate that the GelMA hydrogel was favorable for maintaining the undifferentiated state of hESCs-derived CD34^+^ HSPCs in vitro.

### Establishment of human hematopoietic organoids in GelMA hydrogel with niche-related cells

**Fig. 2:**
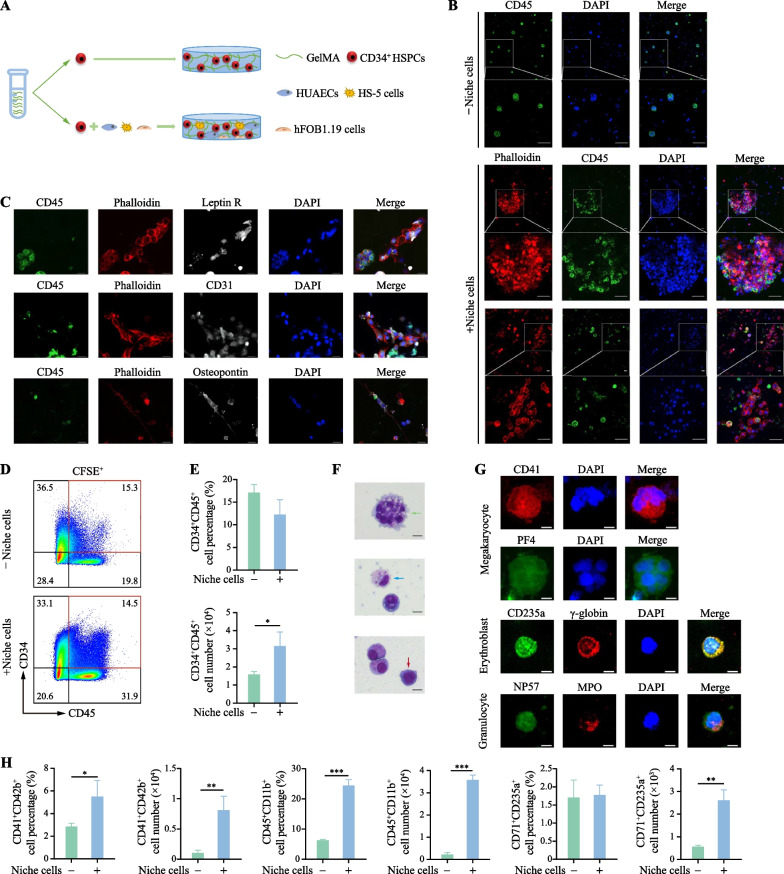
Construction of the in vitro hematopoietic organoids. **a** Schematic diagram of the construction of the hematopoietic organoids. **b** Immunofluorescence images of cells in GelMA under different conditions (scale bar, 50 µm). **c** Various niche cell-specific protein immunofluorescence staining in GelMA with niche cell groups (scale bar, 20 µm). **d** Flow cytometry analysis of CD34 and CD45 expression in cells for each condition. **e** Percentage and number of CD34^+^CD45^+^ cells on day 7 for each condition. Results are presented as the mean ± SD from three independent experiments. Unpaired Student’s t-test, *p < 0.05. **f** Giemsa staining of megakaryocytes, promyelocytes, and erythroblasts from GelMA with niche cells group on day 7 (green: megakaryocytes; blue: promyelocytes; red: erythroblasts. scale bar, 10 µm). **g** Immunofluorescence staining showed the expression of specific proteins associated with megakaryocytes (CD41 and PF4), erythroid cells (CD235a and γ-globin), and granulocytes (NP57 and MPO) when co-cultured with niche cells. Scale bars, 10 μm. **h** Percentage and number of CD41^+^CD42b^+^, CD45^+^CD11b^+^ and CD71^+^CD235a^+^ cells on day 7 for each condition. Results are presented as the mean ± SD from three independent experiments. Unpaired Student’s t-test, *p < 0.05; **p < 0.01; ***p < 0.001

To accurately simulate the complicated BM hematopoietic microenvironment in GelMA in vitro, the GelMA hydrogel was supplemented with several types of BM niche-related cells, including HS-5 cells, HUAECs, and hFOB1.19 cells, to support hematopoietic cell survival, proliferation, and differentiation [14–17]. HS-5 cells, HUAECs, and hFOB 1.19 cells and hESCs-derived CD34^+^ cells were seeded into GelMA hydrogel at a ratio of 1:1:1:3 (Fig. 2a). Notably, hESCs-derived CD45^+^ cells exhibited adherent growth on hematopoietic niche cells (+ Niche cell group), whereas CD45^+^ cells on GelMA without niche cells (− Niche cell group) showed single- or spheroid-like growth after 7 days of culture (Fig. 2b). Moreover, HS-5 cells, HUAECs, and hFOB1.19 cells normally expressed characteristic proteins in this culture environment (Fig. 2c). Additionally, although there was no significant difference in the proportion of CD34^+^CD45^+^ HSPCs between the two groups, the presence of niche cells in GelMA resulted in a higher number of CD34^+^CD45^+^ HSPCs than in their absence (Fig. 2d, e), indicating that niche cells support the proliferation of CD34^+^CD45^+^ HSPCs. The CFU assay results showed that the total number of colonies significantly increased in the + Niche cell group after seven days of culture (Additional file 6: Supplementary Fig. 1). Of note, Wright-Giemsa staining demonstrated the presence of megakaryocytes, promyelocytes and erythroblasts in these organoids (Fig. 2f). Consistently, immunostaining results showed large polyploid megakaryocytes with PF4 protein expression, granulocytes with NP57 and MPO expression, and CD235^+^ erythroid cells with γ-globin protein expression (Fig. 2g). Compared to the -Niche cell group, the + Niche cell group exhibited a higher quantity of megakaryocytic (CD41^+^CD42b^+^), myeloid (CD45^+^CD11b^+^), and erythroid (CD71^+^CD235a^+^) cells (Fig. 2h). These results indicate that the integration of niche cells into GelMA creates a more conducive microenvironment for the formation of hematopoietic organoids, thereby enhancing the proliferation and multilineage differentiation capabilities of hESCs-derived HSPCs.

### The hematopoietic organoids exhibit radiation-induced hematopoietic cell injury effect

**Fig. 3:**
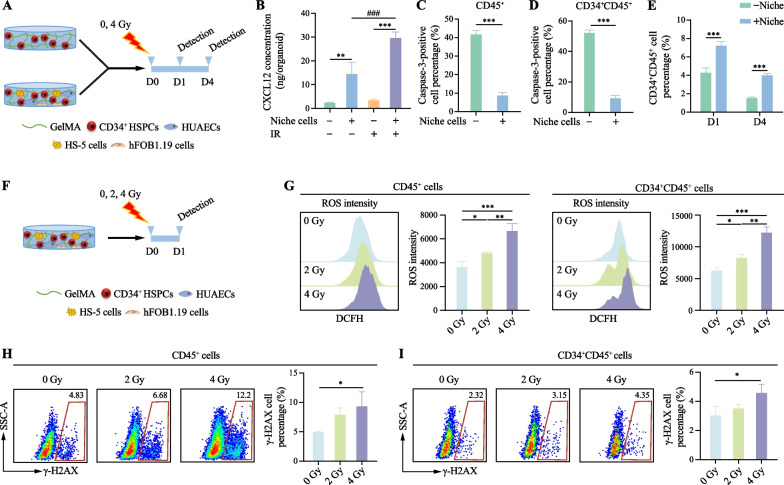
Construction of the in vitro radiation injury model based on the hematopoietic organoids. **a** Schematic diagram demonstrates the organoids from -Niche cell and + Niche cell groups received radiation at 0 or 4 Gy. **b** Comparative analysis of CXCL12 concentration in the two groups under different conditions one day after radiation exposure. Results are presented as the mean ± SD from three independent experiments. One-way ANOVA, **p < 0.01; ***p < 0.001;###*p *< 0.001. **c** Flow cytometry analysis of the percentage of caspase-3-positive cells in CD45^+^ cell population from the two groups one day after radiation exposure. Results are presented as the mean ± SD from three independent experiments. One-way ANOVA, ***p < 0.001. **d** Flow cytometry analysis of the percentage of caspase-3-positive cells in CD34^+^CD45^+^ cell population from the two groups one day after radiation exposure. Results are presented as the mean ± SD from three independent experiments. One-way ANOVA, ***p < 0.001. **e** Percentage of CD34^+^CD45^+^ cells on the first and fourth day after 4 Gy irradiation. Results are presented as the mean ± SD from three independent experiments. One-way ANOVA, ***p < 0.001. **f** Schematic diagram of the construction of the radiation injury model. **g** ROS levels in CD45^+^ and CD34^+^CD45^+^ cells under different radiation doses. Results are presented as the mean ± SD from three independent experiments. One-way ANOVA, *p < 0.05; **p < 0.01; ***p < 0.001. **h** γ-H2AX expression levels in CD45^+^ cells under different radiation doses. Results are presented as the mean ± SD from three independent experiments. One-way ANOVA, *p < 0.05. **I.** γ-H2AX expression levels in CD34^+^CD45^+^ cells under different radiation doses. Results are presented as the mean ± SD from three independent experiments. One-way ANOVA, *p < 0.05

To assess the impact of niche cells in GelMA on HSPCs in radiation damage, organoids were exposed to 4 Gy doses of ionizing radiation (IR) in GelMA with and without niche cells (Fig. 3a). Higher protein concentration of CXCL12 is found in the + Niche cell group compared to those in the -Niche cell group, suggesting that niche cells may interact with hematopoietic cells through chemokine/chemokine receptor axis (Fig. 3b). Previous studies have reported an increase in CXCL12 levels in the BM niche after radiation exposure [30, 31]. We further employed ELISA to measure CXCL12 levels in the organoids before and after radiation. Notably, radiation significantly increased CXCL12 protein concentration in the + Niche cell group (Fig. 3b), a similar response with in vivo bone marrow tissue before and after irradiation. However, the organoids in –Niche cell group exhibited no obvious alteration on CXCL12 protein concentration between non-irradiated and irradiated conditions (Fig. 3b). Furthermore, compared to the -Niche cell group, the + Niche cell group showed a notable reduction in the percentage of caspase-3-positive cells at day 1 post-radiation and significant increases in CD34^+^CD45^+^ cell percentages at day 1 and day 4 after radiation damage (Fig. 3c–e). These findings reflect the potential protective effects of niche cells on HSPCs during the radiation injury process.

To further evaluate whether human hematopoietic organoids have different responses at different doses of IR, the organoids formed in GelMA with niche cells were exposed to IR at doses of 2 and 4 Gy (Fig. 3f). Notably, we found that IR induced dose-dependent increases in ROS-positive cell percentages within the CD45^+^ cell population at day 1 after exposure (Additional file 7: Supplementary Fig. 2). Concurrently, flow cytometry analysis results further demonstrated that IR induced a progressive increase in oxidative stress damage in CD45^+^ and CD34^+^CD45^+^ cells, as evidenced by elevated ROS levels with increased radiation doses (Fig. 3g). Moreover, exposure to a 4 Gy dose of IR significantly augmented the proportion of γ-H2AX-positive cells in CD45^+^ and CD34^+^CD45^+^ populations (Fig. 3h, i), indicating that the hematopoietic organoid can be injured at DNA levels after high dose of radiation.

**Fig. 4:**
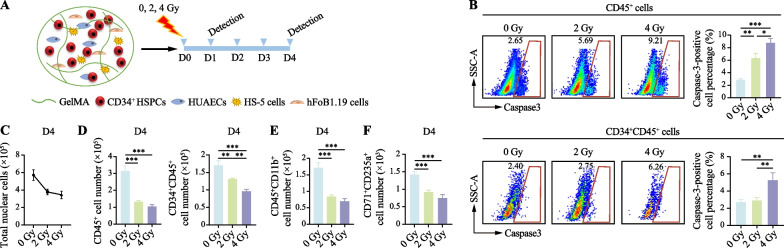
The hematopoietic organoids exhibit radiation-induced hematopoietic cell injury effect. **a** Schematic diagram of apoptosis detection in the radiation injury model after exposure to different doses on the first and fourth days. **b** Flow cytometry analysis of the percentage of caspase-3-positive cells in CD45^+^ and CD34^+^CD45^+^ cells under different radiation doses. Results are presented as the mean ± SD from three independent experiments. One-way ANOVA, *p < 0.05; **p < 0.01; ***p < 0.001. **c** Flow cytometry analysis of the total number of nuclear cells under different radiation doses. **d** Flow cytometry analysis of the number of CD45^+^ and CD34^+^CD45^+^cells under different radiation doses. Results are presented as the mean ± SD from three independent experiments. One-way ANOVA, **p < 0.01; ***p < 0.001. **e** Flow cytometry analysis of the number of CD45^+^CD11b^+^ cells under different radiation doses. Results are presented as the mean ± SD from three independent experiments. One-way ANOVA, ***p < 0.001. **f** Flow cytometry analysis of the number of CD71^+^CD235a^+^ cells under different radiation doses. Results are presented as the mean ± SD from three independent experiments. One-way ANOVA, ***p < 0.001

Subsequently, we evaluated apoptosis in hematopoietic organoids one day after irradiation exposure by assessing caspase-3 expression (Fig. 4a). Remarkably, radiation with 2 and 4 Gy significantly elevated the percentage of caspase-3-positive cells in CD45^+^ cells by 1.22- and 2.07-fold, respectively, compared to that in the non-irradiated group (Fig. 4b). Additionally, in CD34^+^CD45^+^ cells, the percentage of caspase-3-positive cells increased by 0.07- and 0.94-fold, respectively (Fig. 4b). The overall cell count on the fourth day post-irradiation showed that compared with the non-irradiated group, the cell count decreased by approximately 33% and 39% after 2 Gy and 4 Gy irradiation, respectively, aligning with the radiation sensitivity observed in human studies [32] (Additional file 4: Supplementary Fig. 3). In addition, the numbers of blood cells (CD45^+^), HSPCs (CD34^+^CD45^+^), myeloid cells (CD45^+^CD11b^+^), and erythrocytes (CD71^+^CD235a^+^) decreased by approximately 68%, 43%, 59%, and 47% respectively after 4 Gy irradiation, showing relatively severe cytotoxic effects (Fig. 4c–e). These findings highlight the susceptibility of hematopoietic organoids to radiation-induced damage.

### Hematopoietic organoids post-radiation can be used to evaluate the radiation-mitigating effects of G-CSF

**Fig. 5:**
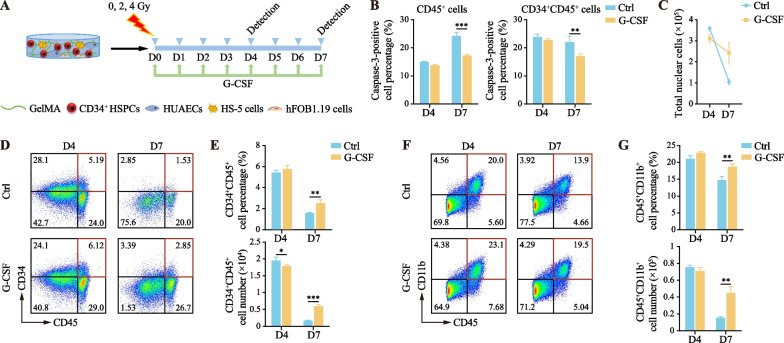
G-CSF exhibits a radiation-mitigating effect in hematopoietic cells. **a** Schematic diagram for effect of G-CSF on hematopoietic cells after radiation exposure. **b** Flow cytometry analysis of the percentage of caspase-3-positive cells in CD45^+^ and CD34^+^CD45^+^ cells in the radiation injury model on the fourth and seventh days following the administration of G-CSF after irradiation. Results are presented as the mean ± SD from three independent experiments. Two-way ANOVA, **p < 0.01; ***p < 0.001. **c** Flow cytometry analysis of the number of total nuclear cells in the radiation injury model on the fourth and seventh days following the administration of G-CSF after radiation. **d–e** Flow cytometry analysis of the percentage and number of CD34^+^CD45^+^ cells in the radiation injury model on the fourth and seventh days following the administration of G-CSF after radiation. Results are presented as the mean ± SD from three independent experiments. Two-way ANOVA, *p < 0.05; **p < 0.01; ***p < 0.001. **f**–**g** Flow cytometry analysis of the percentage and number of CD45^+^CD11b^+^ myeloid cells in the radiation injury model on the fourth and seventh days following the administration of G-CSF after radiation. Results are presented as the mean ± SD from three independent experiments. Two-way ANOVA, **p < 0.01

G-CSF is a myeloid growth factor considered as an ideal medication for reducing radiation-induced BM suppression [33]. Following the administration of G-CSF after 4 Gy irradiation in hematopoietic organoids (Fig. 5a), a significant reduction in the percentage of caspase-3-positive cells within both CD45^+^ and CD45^+^CD34^+^ cells was observed by the seventh day (Fig. 5b). Simultaneously, from the fourth to the seventh day of culture, the cell counts in the G-CSF treated group decreased by approximately 22%, in stark contrast to a 70% reduction in the control group (Fig. 5c). Furthermore, after G-CSF addition, there was a significant increase in the proportion and number of CD45^+^CD34^+^ HSPCs and CD45^+^CD11b^+^ myeloid cells (Fig. 5d–g), which is consistent with previous findings that G-CSF stimulates the proliferation of HSPCs and induces myeloid cell differentiation, thus ameliorating acute damage to the hematopoietic system caused by radiation [34, 35]. These findings demonstrate the effectiveness of G-CSF in safeguarding blood cells after radiation injury, and reflect the utility of hematopoietic organoids in assessing therapeutic strategies for the mitigating and rehabilitation of bone marrow radiation damage.

## Discussion

In this study, we aimed to establish hematopoietic organoids using hESCs-derived HSPCs, niche-related cells, and a GelMA hydrogel and to evaluate their utility for modeling hematopoietic injury and regeneration. Although various tissue-specific organoids have been generated and applied in the biomedical field, establishing human hematopoietic organoids or BM on-a-chip models remains a significant challenge because of the inherent complexity of BM tissues. HSPCs are the key seed cells in the production of hematopoietic organoids. However, it is difficult to obtain rare HSPCs from mobilized peripheral blood, BM, or a single unit of cord blood. hPSCs, such as hESCs, are ideal seed cells for obtaining large numbers of HSPCs and for scalable manufacturing of human hematopoietic or BM organoids. Accumulating evidence demonstrates that hESCs can differentiate into HSPCs and multilineage blood cells [36, 37]. Therefore, we used a stepwise induction protocol to generate human CD34^+^ HSPCs from hESCs.

The manufacture of organoids often requires the use of Matrigel or hydrogels to provide a 3D biomimetic microenvironment [38, 39]. Among various hydrogels, GelMA has been widely applied in 3D cell culture and has demonstrated its capacity to effectively simulate the biophysical properties of the ECM and promote cell adhesion and growth [40]. GelMA possesses an arginine-glycine-aspartic acid (RGD) sequence and a target sequence for matrix metalloproteinases (MMPs) and can be modified according to different requirements [41]. In recent years, GelMA and other types of hydrogels have been used to support HSPC growth and expansion, demonstrating the reliability of these hydrogels for 3D hematopoietic organoid modeling. Notably, we found that the GelMA hydrogel as a 3D scaffold could support hESCs-derived HSPC survival and maintain an undifferentiated state. An increasing number of studies have demonstrated that in vitro culture of HSPCs causes non-physiological oxidative stress injury and leads to a decreased self-renewal capacity of HSCs [42, 43]. Notably, our 3D culture system based on the GelMA hydrogel has the advantage of reducing ROS levels in HSPCs, which supports its role in maintaining HSPC function. The maintenance of adult hematopoietic function requires BM niche cells that can regulate self-renewal, proliferation, and differentiation of HSCs [44–47]. Various niche-related cell lines with characteristics of endothelial cells, stromal cells, or osteoblasts have been used to support HSPC function or to construct BM-on-a-chip [14]. Based on the complicated characteristics of the BM niche, we designed and established a hematopoietic organoid model consisting of various niche-related cells and GelMA hydrogel. These cells provide crucial signaling molecules like CXCL12 chemokine, Leptin receptor, and osteopontin, essential for HSC self-renewal, proliferation, and differentiation [48, 49]. Moreover, the evident adherence between the niche and the hematopoietic cells reflect the significance of intercellular interactions. The human hematopoietic organoids that we constructed maintained HSPC function and produced myeloid cells, megakaryocytes, and erythroid cells, indicating that this model can recapitulate normal and injury-conditioned human hematopoiesis in vitro.

The similarity of the cell composition and function of our hematopoietic organoids to human BM prompted us to develop a hematopoietic disease model and assess the drug response. The hematopoietic system is highly sensitive to radiation and other chemical factors. Many laboratories are devoted to screening and developing new drugs to protect the hematopoietic system and mitigate hematopoietic injury. However, the lack of scalable human BM organoid platforms has significantly hampered the study of radiation-stressed hematopoiesis and evaluation of novel therapies. We tested and proved that biomimetic hematopoietic organoids show radiation dose-dependent injury responsiveness in hematopoietic cells as evidenced by increased ROS levels and DNA damage degree in them. Accumulated studies indicated that low levels of ROS are essential for maintaining HSC self-renewal whereas radiation induced high ROS level in BM niche that caused HSPC damage [50]. As ROS are known to be involved in hematopoietic injury [51–53], it would be meaningful to further investigate the dynamic change of ROS in different types of hematopoietic and niche cells in the organoids after radiation.

We further tested the responsiveness of this radiation-injured hematopoietic organoid to G-CSF, an FDA-approved radiation countermeasure. We found that G-CSF reduced hematopoietic cell apoptosis and promoted myeloid cell regeneration in the organoids after 4 Gy radiation, which is consistent with its known functions in vivo [35, 54, 55]*.* Considering that G-CSF can exacerbate radiation-induced long-term BM injury and induce cell senescence [56, 57], more work needs to be done for us to further evaluate the various roles of G-CSF, including extending the culture period of the hematopoietic organoids after radiation and increasing detection criteria after long-term in vitro culture. Additionally, it is worthy to investigate whether the hematopoietic organoids can be used to screen or test HSPC mobilization or homing agents, such as G-CSF, Me6TREN or TPO [58, 59].

## Conclusions

Our data demonstrated that 3D hematopoietic organoids composed of HSPCs, multilineage blood cells, and niche-related cells were successfully constructed in vitro. These organoids retained their capacity for HSPCs proliferation and multi-lineage differentiation in vitro. In addition, hematopoietic organoids can produce an organ-level response to radiation toxicity and successfully recapitulate regenerative responses to the radiation countermeasure drug G-CSF. Our study supports the standard and scalable manufacturing of human hematopoietic organoids for radiation-injured or other hematopoietic disease models and drug screening or testing.

### Supplementary Information

**Additional file 1:** Supplemental methods.

**Additional file 2: Table S1.** The composition of BEL medium.

**Additional file 3: Table S2.** Antibodies used in this study.

**Additional file 4:** **Table S3.** Medium used in this study.

**Additional file 5:** **Table S4.** Reagents used in this study.

**Additional file 6: Supplemental Figure 1.** Total number of colonies under different culture conditions.

**Additional file 7:** **Supplemental Figure 2**. Immunofluorescence staining assessed CD45 and DCFH expression under different radiation doses and quantified DCFH positivity in CD45^+^ cells (scale bar, 50 µm).

**Additional file 8: Supplemental Figure 3.** Total number of cells under different radiation doses.

## Data Availability

All data generated or analyzed during this study are included in this published article and its supplementary information files.

## Abbreviations

BM: Bone marrow
IR: Ionizing radiation
2D: Two-dimensional
3D: Three-dimensional
GelMA: Gelatin-methacryloyl
hESCs: Human embryonic stem cells
iPSCs: Induced pluripotent stem cells
hPSCs: Human pluripotent stem cells
ROS: Reactive oxygen species
G-CSF: Granulocyte colony-stimulating factor
HSPC: Hematopoietic stem and progenitor cell
ECM: Extracellular matrix
MA: Methacrylic anhydride
RGD: Arginine-glycine-aspartic acid
MMPs: Matrix metalloproteinases
HS-5: Human bone marrow stromal cell line
HUAECs: Human umbilical artery endothelial cells
hFOB 1.19: Human fetal bone osteoblast cell line
bFGF: Basic fibroblast growth factor
BMP4: Bone morphogenetic protein 4
VEGF: Vascular endothelial growth factor
SCF: Stem cell factor
TPO: Thrombopoietin
IL: Interleukin
Flt3L: Flt3-Ligand
LAP: Lithium phenyl-2,4,6-trimethylbenzoylphosphinate
CFSE: Carboxyfluorescein diacetate succinimidyl ester
PF4: Platelet factor 4
NP57: Neutrophil elastase
MPO: Myeloperoxidase
CFU: Hematopoietic colony-forming unit
BFU-E: Burst-forming unit-erythroid
CFU-GM: CFU-granulocyte, monocyte
CFU-GEMM: CFU-granulocyte, erythrocyte, monocyte and megakaryocyte
