# Supplementary material for: Establishment of human hematopoietic organoids for evaluation of hematopoietic injury and regeneration effect
Source: Stem Cell Res Ther. 2024 May 4;15:133. doi: 10.1186/s13287-024-03743-y (PMC11070084; doi:10.1186/s13287-024-03743-y)
Supplement: Supplementary file 1 — Additional file 1: Supplemental methods. [file 13287_2024_3743_MOESM1_ESM.docx]

**Supplemental methods**

**Giemsa staining.** Cells were harvested using GelMA Lysis Buffer (EFL) and washed once with PBS. They were then cytocentrifuged onto glass slides. One drop of Giemsa A solution was added, and cells were stained for 1 minute at room temperature. Subsequently, 3 drops of Wright-Giemsa B solution were added, the slides were gently shaken to ensure full mixing, and cells were stained for an additional 10 minutes at room temperature. After staining, the slides were gently rinsed with water, and the cells were observed and photographed under a light microscope.

**Detection of intracellular ROS level.** Cells were harvested using GelMA Lysis Buffer (EFL) and washed once with PBS. They were then incubated with 10 µM DCFH-DA in PBS for 30 minutes at 37°C in the dark to stain the cells. Post-staining, the cells were analyzed using a BD FACS Aria II flow cytometer (BD Biosciences, Franklin Lakes, NJ, USA). Data from the flow cytometry were processed and analyzed with FlowJo software (TreeStar, Ashland, OR, USA).

**Detection of γ-H2AX and Caspase-3 expression in cells.** After incubation with all membrane surface antibodies, cells were fixed with Cell Fixation and Permeabilization Solution at room temperature for 10 minutes and then washed twice with BD Perm/Wash™ Buffer. The cells were then resuspended in BD Perm/Wash™ Buffer, and anti-γ-H2AX and anti-Caspase-3 antibodies were added for incubation at 4°C overnight. Following staining, the cells were analyzed using a BD FACS Aria II flow cytometer (BD Biosciences, Franklin Lakes, NJ, USA). Data analysis was performed using FlowJo software (TreeStar, Ashland, OR, USA).

**Supplemental Figure 1. Total number of colonies under different culture conditions.**

**Supplemental Figure 2. Immunofluorescence staining assessed CD45 and DCFH expression under different radiation doses and quantified DCFH positivity in CD45^+^ cells (scale bar, 50 µm)**.

**Supplemental Figure 3. Total number of cells under different radiation doses.**
