# Supplementary figures and images for: Establishment of human hematopoietic organoids for evaluation of hematopoietic injury and regeneration effect
Source: Stem Cell Res Ther. 2024 May 4;15:133. doi: 10.1186/s13287-024-03743-y (PMC11070084; doi:10.1186/s13287-024-03743-y)

**Figure S1**

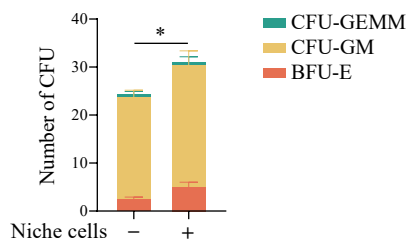

Supplement: Supplementary file 6 — Additional file 6: Supplemental Figure 1. Total number of colonies under different culture conditions. [file 13287_2024_3743_MOESM6_ESM.pdf]

Figure S2

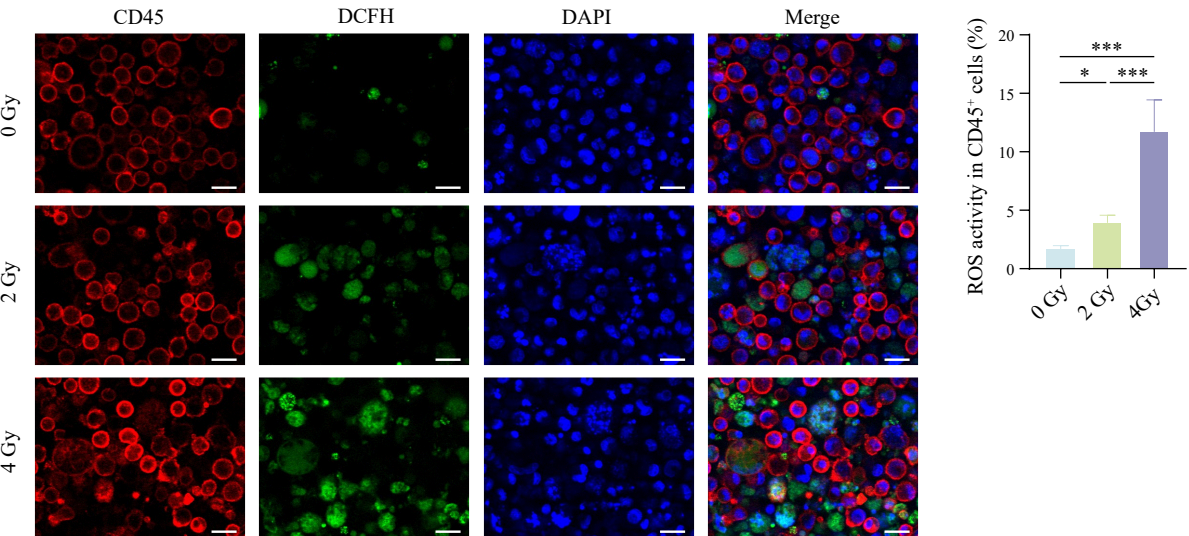

Supplement: Supplementary file 7 — Additional file 7: Supplemental Figure 2. Immunofluorescence staining assessed CD45 and DCFH expression under different radiation doses and quantified DCFH positivity in CD45+ cells (scale bar, 50 µm). [file 13287_2024_3743_MOESM7_ESM.pdf]

**Figure S3**

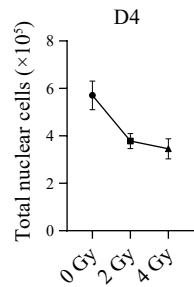

Supplement: Supplementary file 8 — Additional file 8: Supplemental Figure 3. Total number of cells under different radiation doses. [file 13287_2024_3743_MOESM8_ESM.pdf]
